# Score-based generative diffusion models to synthesize full-dose FDG brain PET from MRI in epilepsy patients

**DOI:** 10.3389/frai.2026.1841677

**Published:** 2026-06-16

**Authors:** Jiaqi Wu, Jiahong Ouyang, Farshad Moradi, Mohammad Mehdi Khalighi, Greg Zaharchuk

**Affiliations:** Radiology Department, Stanford University, Stanford, CA, United States

**Keywords:** artificial intelligence, epilepsy, image translation, score-based generative model, transformer

## Abstract

Fluorodeoxyglucose (FDG) PET to evaluate patients with epilepsy is one of the most common applications for simultaneous PET/MRI, given the need to image both brain structure and metabolism but is suboptimal due to the radiation dose in this young population. Little work has been done synthesizing diagnostic quality PET images from MRI data or MRI data with ultralow-dose PET using advanced generative AI methods, such as diffusion models, with attention to clinical evaluations tailored for the epilepsy population. We compared the performance of diffusion- and non-diffusion-based deep learning models for the MRI-to-PET image translation task for epilepsy imaging using simultaneous PET/MRI in 52 subjects (40 train/2 validate/10 hold-out test). We tested three different models: 2 score-based generative diffusion models (SGM-Karras Diffusion [SGM-KD] and SGM-variance preserving [SGM-VP]) and a Transformer-U-net. We report results on standard image processing metrics as well as clinically relevant metrics, including congruency measures (Congruence Index and Congruency Mean Absolute Error) that assess hemispheric metabolic asymmetry, which is a key part of the clinical analysis of these images. We compared the model performance using different inputs such as T1-weighted (T1w), T2 FLAIR (T2F), and 1% ultralow-dose PET images to evaluate the effect and necessity of each imaging contrast. The SGM-KD produced the best qualitative and quantitative results when synthesizing PET purely from T1w and T2 FLAIR images with the least mean absolute error in whole-brain specific uptake value ratio (SUVR) and highest intraclass correlation coefficient. When 1% low-dose PET images are included in the inputs, all models improve significantly and are interchangeable for quantitative performance and visual quality. SGMs hold great potential for pure MRI-to-PET translation, while all 3 model types can synthesize full-dose FDG-PET accurately using MRI and ultralow-dose PET. This suggests that deep learning diffusion models could reduce or eliminate radiation dose for patients being evaluated for epilepsy.

## Introduction

Epilepsy, a prevalent neurological disorder characterized by recurrent seizures, necessitates precise diagnostic approaches to ensure effective management. ^18^F-fluoro-2-deoxyglucose (^18^F-FDG) Positron Emission Tomography (PET) imaging of brain glucose metabolism is a well-established technique for localizing epileptogenic foci. PET provides functional information by detecting metabolic changes, which are crucial for localizing epileptogenic zones (EZs), especially in cases where Magnetic Resonance Imaging (MRI) findings are inconclusive. Meanwhile, MRI is an important diagnostic tool, offering detailed anatomical images and enabling the identification of structural abnormalities such as mesial temporal sclerosis and focal cortical dysplasias that may underlie epileptic activity (Cendes et al., 2016). A multi-modal approach integrating the functional imaging of FDG-PET with the morphologic information from MRI in presurgical evaluation of epilepsy has been shown to improve outcomes (Ponisio et al., 2021). One study also suggests that diagnostic accuracy for EZ detection in focal epilepsy may be higher for FDG-PET/MRI compared with FDG-PET/CT (Kikuchi et al., 2021).

However, obtaining PET images poses several challenges. PET scanners are far less accessible than MRI scanners, particularly in middle- and low-income countries and rural locations. Even in high-income countries, access to PET often involves long-distance travel and significant expenses (Gallach et al., 2020). Moreover, FDG-PET involves the injection of a radioactive tracer into the body, with the added absolute risk of subsequent malignancy from FDG-PET/CT that has been estimated at 0.5–0.6% (Huang et al., 2009). In contrast, MRI is a widely available imaging technique that does not involve ionizing radiation. Therefore, exploring the feasibility of synthesizing FDG-PET images from multi-contrast MRI images or MRI plus lower dose FDG-PET is a worthwhile endeavor.

Advancements in PET imaging have explored both hardware and algorithm-based strategies to improve image quality and reduce dose. The kernel-based reconstruction, for instance, assumes that voxels with similar features should also have similar PET uptake values (Deidda et al., 2019). For hardware advancements, next-generation scanners such as NeuroEXPLORER (Li et al., 2024) have been shown to enable lower injected doses and count levels, although not approaching the 1% dose investigated in this work. Moreover, such systems remain less accessible and substantially more expensive than conventional clinical PET scanners. While these hardware and reconstruction improvements are important, artificial intelligence has shown impressive performance for medical image translation. U-net models using nested convolution neural networks (CNNs) represent some of the earliest work in this area (Xiang et al., 2017). More recently, Generative Adversarial Networks (GANs) have been added to the basic U-net structure to improve performance. For instance, MedGAN (Armanious et al., 2020) was developed for conversion of PET to CT images. A Swin-Transformer based GAN (Yan et al., 2022) was used for multi-sequence MRI translation, while Transformer-U-net models have shown promise for MRI-to-PET translation and medical image segmentation (Ouyang et al., 2024; Chen J. et al., 2021). The advances in diffusion-based models such as Diffusing Denoising Probabilistic Models (DDPM) have been explored by prior work (Jiang et al., 2024; Lyu and Wang, 2022; Singh et al., 2024; Gong et al., 2024) in multi-contrast MRI sequence-to-sequence translation, CT-to-MRI translation, and PET reconstruction, demonstrating better performance than CNN and GAN models. The use of AI-based denoising software, as demonstrated in this study, provides a complementary and uniquely scalable approach to enhancing PET image quality.

Although there is abundant literature leveraging 2D models for 3D volumes in the domain of medical imaging, MRI-to-PET translation using generative models in the context of epilepsy remain largely under-explored, in part due to the lack of large public datasets. Non-epilepsy public MRI and PET datasets also suffer from the lack of access to list-mode raw PET data to allow for realistic synthesis of low-dose images. Additionally, current image processing evaluation metrics such as structural similarity (SSIM), peak signal-to-noise ratio (PSNR), and root mean squared error (RMSE), which are frequently used in natural image assessments, are not uniformly applicable to medical images. In the context of PET synthesis, for instance, accuracy for measuring the exact standardized uptake value (SUV) of a region may be of lesser importance than ensuring that the relative intensity with respect to a reference region (i.e., the SUV ratio [SUVR]) matches that of the acquired image. More importantly for epilepsy patients, these metrics may fail to capture clinically relevant indicators such as the hemispheric asymmetry in metabolism, which is important for epilepsy diagnosis and classification.

In this study, we compare state-of-the-art score-based generative and transformer models for synthesizing full-dose FDG-PET from MRI alone (cross-modality translation) and from MRI combined with ultralow-dose FDG-PET (PET denoising) in epilepsy patients. Using 2D models, we go beyond earlier works that focused only on T1-weighted (T1w) images, also incorporating other images, such as T2 FLAIR and 1% ultralow-dose PET as training inputs, demonstrating the potential for reduced radiation exposure. Finally, we propose and evaluate the models on metrics with clinical relevance for epilepsy patients that assess the fidelity of representing hemispheric metabolic asymmetries.

## Materials and methods

### Diffusion models in medical imaging

DDPMs have captured significant attention in recent years as a class of generative models that generate data by learning to reverse a diffusion process. These models operate in two stages: in the forward process, the original data is gradually corrupted with Gaussian noise over multiple timesteps, eventually becoming pure noise. In the backward process, the model learns to reverse this transformation step-by-step, recovering the original data distribution from the noise. Conditional inputs such as prior or other images and textual context can be passed into the network for all timesteps. This framework has been actively explored in medical imaging due to its ability to capture complex data distributions. For instance, a DDPM with fast training and sampling was applied to T1w-to-T2-weighted image translation using the Brain Tumor Segmentation (BraTS) dataset (Jiang et al., 2024). Additionally, DDPMs have shown promise in CT-to-MRI image translation (Lyu and Wang, 2022) and for PET denoising (Chen K. T. et al., 2021; Singh et al., 2024; Gong et al., 2024). However, based on our initial exploration, DDPMs / Latent Diffusion models did not produce stable/satisfactory results with small datasets.

Closely related to DDPMs, Score-Based Generative Models (SGMs) learn the gradient of the log probability density function of the data of interest, known as the score function. SGMs leverage stochastic differential equations (SDEs) to disturb, model, and sample from the data distribution, using sampling techniques such as Predictor–Corrector, Euler-Maruyama, and Heun’s methods (Lyu and Wang, 2022; Singh et al., 2024; Gong et al., 2024; Song et al., 2021; Karras et al., 2022).

In the forward process in Equation 1, the data distribution is disturbed toward Gaussian noise, where change of x at time t ( $dx_{t}$ ) consists of a drift term *f(x, t)*, a diffusion term *g(t)*, and a Weiner process term $dw_{t}$ (white noise) (Song et al., 2021). The drift term f can be understood as the deterministic part of the system’s evolution, while the diffusion term g * dw_t_ resembles the stochastic component adding random noise to the system.


$$\mathrm{dx}=f(x,t)+g(t)dw_{t}$$ (1)

In the backward process in Equation 2, the Gaussian noise is gradually removed from the original data following the backward SDE Equation (17). A deep learning network with parameters θ is used to learn the gradient of the log probability of the data distribution $,\nabla_{x}\log p_{t}(x)$ , and is referred to as the score $s_{\theta }(x,t)$ in Equation 3. Once this gradient is learned, we can obtain our target image from repetitively removing noise through this backward process.


$$\mathrm{dx}=[f(x,t)-g{\left(t\right)}^{2}\nabla_{x}\log p_{t}(x)]\mathrm{dt}+g(t)dw_{t}$$ (2)

$$\nabla_{x}\log p_{t}(x|x_{0})=s_{\theta }(x,t)\;$$ (3)

Various score-based models have been used in medical image tasks. SGM with Variance Exploding SDE (VESDE) has been applied to CT-to-MRI image translation and achieved better performance than CNN and GAN models for SSIM and PSNR (Lyu and Wang, 2022). SGM with Variance Preserving SDE (VPSDE) has been applied to PET reconstruction (Singh et al., 2024), incorporating the use of forward projections of PET images to count measurement into its sampling. Next, we introduce the SGM we used in this work in more detail.

### Conditional SGM

VPSDE was used in (Singh et al., 2024) for a PET reconstruction task and was selected for evaluation in the current work. VPSDE is a type of SDE with the formulation in Equation 4 during the forward pass, where β(t) is a positive noise scalar with 0 < $\beta_{1},\beta_{2},\ldots \beta_{T}<1\;[17]$ (Song et al., 2021). At time t, the noise-disturbed x follows a Normal distribution with mean of $x(0)e^{-\frac{1}{2}\int_{0}^{t}\beta (s)\mathrm{ds}}$ and variance ${\sigma_{t}}^{2}=I-Ie^{-\int_{0}^{t}\beta (s)\mathrm{ds}}$ (Equation 5), and the theoretical gradient of the disturbed data is formulated in Equation 6, where ϵ is the *true noise* we add in the forward process.


$$\mathrm{dx}=-\frac{1}{2}\beta (t)x.\mathrm{dt}+\sqrt{\beta (t)}.\mathrm{dw}$$ (4)

$$p(x_{t}\;|x_{0})=N(x_{t};x_{0}e^{-\frac{1}{2}\int_{0}^{t}\beta (s)\mathrm{ds}},I-Ie^{-\int_{0}^{t}\beta (s)\mathrm{ds}})$$ (5)

$$\nabla_{x}\log p_{t}(x)=-\frac{x_{t}-x_{0}}{\sigma_{2}^{t}}=-\frac{x_{t}-(x_{t}-\varepsilon \sigma_{t})}{{\sigma_{t}}^{2}}=-\frac{\varepsilon }{\sigma_{t}},\text{where}\;{\sigma_{t}}^{2}=I-Ie^{-\int_{0}^{t}\beta (s)\mathrm{ds}}$$ (6)

This SGM generates PET samples conditioned on the MRI inputs or the MRI inputs plus the PET ultralow-dose input (denoted as *y*), so that the learning of the distribution is conditioned on *y*. We can rewrite Equations 2 and 3 to Equations 7 and 8 with this additional conditioning input *y*. The conditional neural network $s_{\theta }$ aims to learn the true gradient $-\frac{\varepsilon }{\sigma_{t}}$ in the forward process by minimizing the loss function with conditional inputs (scaled by weighting $\lambda_{t}$ ) shown by Equation 9. The loss (L_SGM_) is taken with expectation to all time points and all training data x.


$$\mathrm{dx}=[f(x,t)-g{\left(t\right)}^{2}\nabla_{x}\log p_{t}(x)]\mathrm{dt}+g(t)dw_{t}$$ (7)

$$\nabla_{x}\log p_{t}(x|x_{0},y)=s_{\theta }(x,y,t)\;$$ (8)

$$L_{\mathrm{SGM}}=E_{t}\{\lambda_{t}E_{x_{0}}E_{x_{t}|\;x_{0},y}[\begin{array}{l} \left\|s_{\theta }(x,y,t\right)\\ -\nabla_{x}\log p_{0t}(x_{t}\;|\;x_{0},y)\;{\|}_{2}^{2} \end{array}]\}$$ (9)

Substituting the target theoretical gradient from Equation 6, we get the final loss function below in Equation 10 with known parameters $\sigma_{t}$ (computed from the pre-determined noise scalar β s in Equation 5).


$$L_{\mathrm{SGM}}=E_{t}\{\lambda_{t}E_{x_{0}}E_{x_{t}\;|\;x_{0},y}[{\left\|s_{\theta }(x,y,t)+\frac{\varepsilon }{\sigma_{t}}\right\|}_{2}^{2}]\}$$ (10)

**Figure 1:**
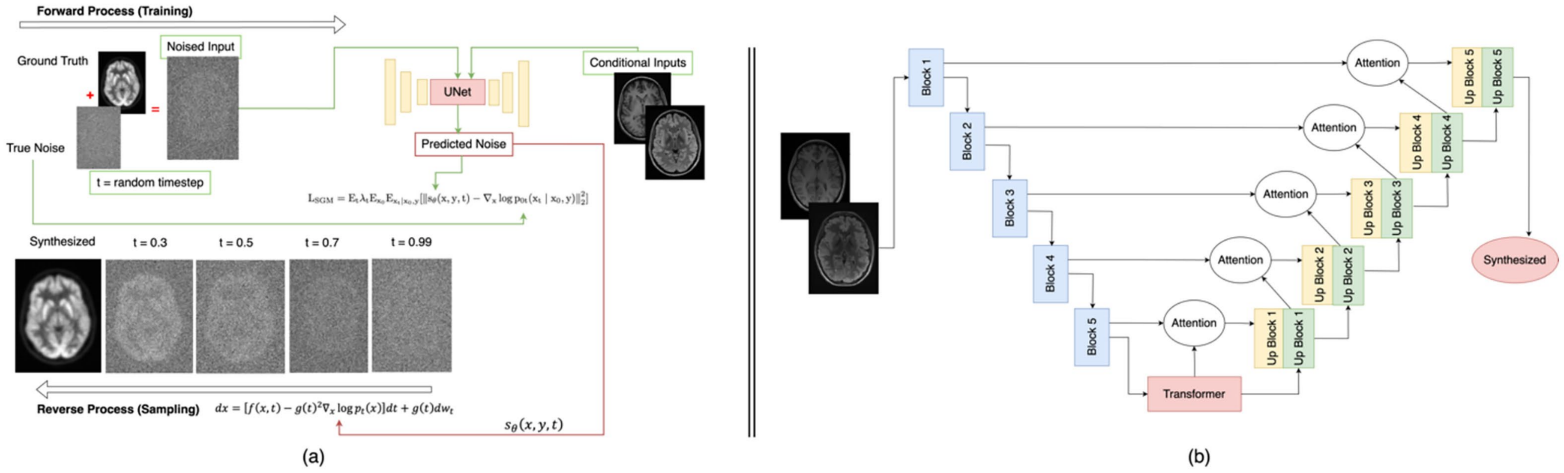
Visualization of SGM and TransUnet architectures. **(a)** In SGM, training occurs on the forward process, where random timesteps + noisy inputs + conditional inputs are used to minimize the loss between true and predicted noise (green boxes/arrows). The denoising process starts from pure random noise. At every timestep *t*, the timestep + noisy predictions + conditional inputs are passed to the model. The model outputs a prediction on the gradient of the data distribution at current timestep, which is used to update the prediction (red arrow). **(b)** In TransUnet, the blue blocks are the downsampling blocks. The yellow and green blocks are different inputs at each layer and are concatenated together for the subsequent upsampling procedure. Channel and spatial attention are also added to each layer.

For the backward sampling process, our sampler follows a predictor and corrector style from Song et al. (2021) with the following algorithm. Starting with $x_{N}$ from Gaussian noise, the predictor uses the score from the network combined with Equation 7 to iteratively update x. After each predictor step, a fixed number of corrector steps use a normalized and scaled score to further improve the current x. The overall workflow of the reverse process is shown in Figure 1a. At each timestep, the noisy prediction, the timestep information, and the conditional inputs are passed to the neural network, which outputs the gradient of the current prediction, which is used for updates and corrections.

Algorithm 1 VPSDE sampling

**Figure:**
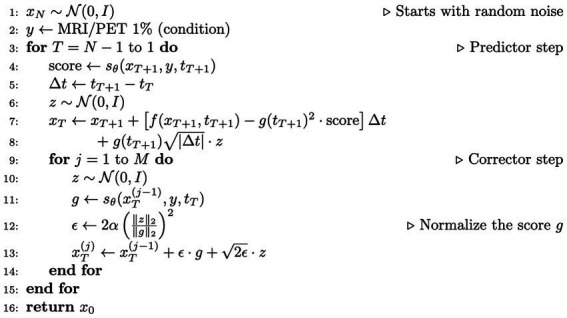

Another proposed architecture using SGM comes from Karras et al. (2022), which we abbreviate as SGM-KD, which envisions a denoiser D reconciling different SDE schemes with the following formulation in Equation 11:


$$D_{\theta }(x,\sigma )=c_{\text{skip}}(\sigma )x+c_{\text{out}}(\sigma )F_{\theta }(c_{\text{in}}(\sigma )x;c_{\text{noise}}(\sigma ))$$ (11)

Here σ is the noise level at a specific timestep. $F_{\theta }$ is the neural network to be trained on noise-disturbed data, $c_{\text{skip}}(\sigma )$ is a σ -dependent scalar modulating the skip connection, $c_{\text{in}}(\sigma )$ and $c_{\text{out}}(\sigma )$ modulates the input and output magnitude, and $c_{\text{noise}}(\sigma )$ is a scalar served as a conditional input into the network. In our task, y is the MRI or MRI + PET ultralow-dose image, and x is the full-dose PET data. We can add our conditions into the equation in Equation 12:


$$D_{\theta }(x,\sigma ,y)=c_{\text{skip}}(\sigma )x+c_{\text{out}}(\sigma )F_{\theta }(c_{\text{in}}(\sigma )x;c_{\text{noise}}(\sigma ),y)$$ (12)

The denoiser aims to minimize the difference between the denoised output with the target x (Equation 13), which can be regarded as predicting and removing the noise added at different steps.


$$\begin{array}{l} \text{Loss}=E_{\sigma ,\mathrm{PET},\text{noise},\mathrm{MRI}}\\ \left[\lambda (\sigma )\|D(\mathrm{PE}T_{\text{clean}}+\text{noise};,\sigma ;\mathrm{MRI})-\mathrm{PE}T_{\text{clean}}\|\right] \end{array}$$ (13)

During the sampling process, we used the paper’s stochastic sampler, which averages the gradient from the current timestep and a future timestep for improved quality:

Algorithm 2 Stochastic sampler with σ(t) = t, s(t) = 1 and conditional denoiser D_θ_

**Figure:**
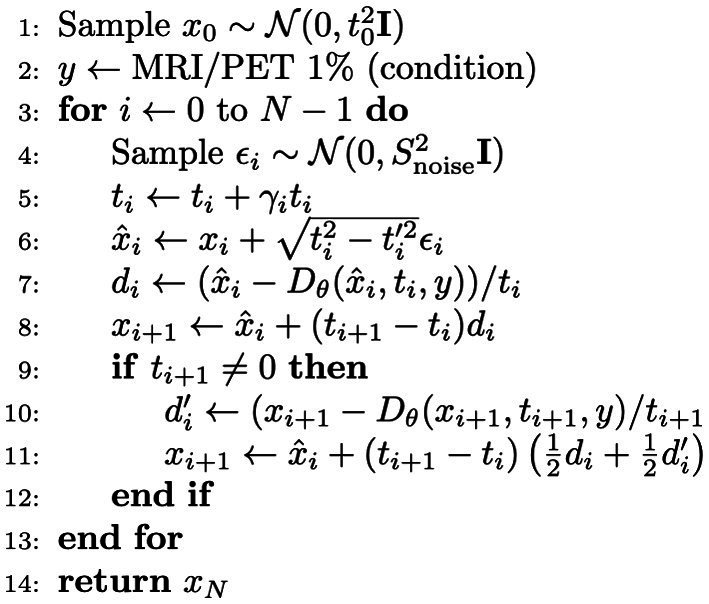

### Transformer-U-net (TransUnet)

TransUnet, as developed in a previous work (Ouyang et al., 2024), takes multi-contrast MRI inputs to predict full-dose FDG-PET, demonstrating strong performance in a brain tumor population. As shown in Figure 1b, the multi-contrast inputs are concatenated at the start of the network. There are 5 blocks with batch-normalization and convolution layers with stride of 2 so that the 256×256 inputs are downsampled to 8×8. Next, a transformer block is applied on the flattened inputs at this innermost layer with multi-head self-attention to capture global information. During the upsampling blocks, spatial attention, channel attention, and upsampling are applied to the inputs and concatenated with the corresponding inputs from the downsampling blocks. Finally, an output convolution layer is applied to obtain the final synthetic PET slice.

### Patient population and imaging methods

**Figure 2:**
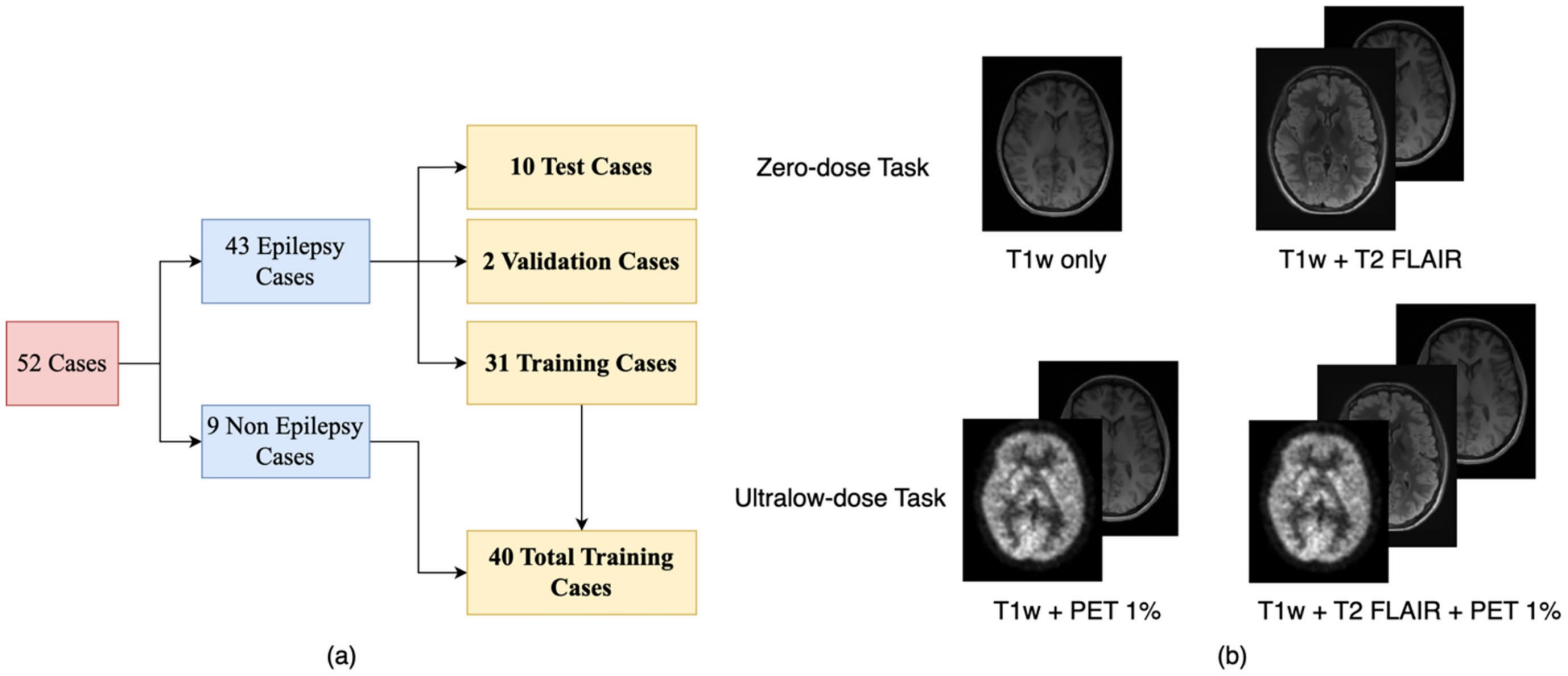
**(a)** Visualization of data split between training, validation, and test sets. **(b)** The 4 different input combinations explored in this study.

After receiving a waiver of consent from our IRB, we identified consecutive adult patients who underwent simultaneous FDG 3.0 T brain PET/MRI (GE Signa, Waukesha, WI) examinations for clinical purposes between August 2022 and October 2024 with available list-mode data. The primary clinical indication for these studies was seizure evaluation, though cases with other indications (e.g., dementia, tumor) were also present. However, only patients undergoing evaluation for epilepsy were included in the test sets, reflecting our specific focus on epilepsy in this study. All available cases, including non-epilepsy cases, were included in the training set to maximize dataset size. Dataset splits and input details are shown in Figure 2.

All patients received a standard full-dose radiotracer, approximately 3.0 MBq/kg. PET counts were acquired for 10 min following a 40 min uptake time. For both the 100% and ultralow-dose PET images, the reconstruction method was ordered subset expectation maximization using 28 subsets and 4/1 iterations. A 4 mm Gaussian smoothing filter was applied to all images. Ultralow-dose PET images were created using list-mode random count under-sampling. Specifically, for all coincidence events recorded in the list-mode data, we selected 1 event every 100 events for 1% PET simulation and passed the selected events into the 3D PET reconstruction (Chen et al., 2020). The dimensions of each reconstructed PET was 256 × 256 × 89 with nominal resolution of 1.2 × 1.2 × 2.78 mm. High-resolution 3D T1w and T2 FLAIR images were acquired using our clinical protocol with the following parameters: T1: TR 6–8.5 ms, TI = 400–600 ms, TE = 2.8 ms, flip angle = 12°, slice thickness = 1.5 mm; T2 FLAIR: TR 5,602–6,002 ms, TI = 1,567–1,765 ms, TE = 127 ms, flip angle = 90°, slice thickness = 2 mm.

### Model implementation details

We evaluated a TransUnet and the 2 SGMs introduced earlier due to their stable synthetic quality and efficient sampling. Specifically, we selected SGM-VP (Singh et al., 2024) and SGM-KD (Karras et al., 2022), as described above. For the SGMs, the data is normalized to a range of [0, 1] for SGM-VP and [−1, 1] for SGM-KD. Additional hyperparameters included 100–300 sampling steps for noise removal, a minimum noise level of 0.1 and a maximum noise level of 10. For TransUnet, we follow the architecture described in (Ouyang et al., 2024) and normalized by dividing all voxel values by the mean value of the volume. All MRI volumes were co-registered to PET in native space to avoid information loss. More details on the training hyperparameters can be found in Supplementary material.

We investigated the effect of multi-sequence MRI and ultralow-dose PET using 4 different input combinations for each model: T1w only (no PET data), T1w + T2-FLAIR (no PET data), and 1% PET data combined with either T1w or T1w + T2-FLAIR (Figure 2b). For all models, we used 3 MRI slices to predict the middle PET slice. Larger numbers of surrounding slices were explored but showed limited improvement. Training was done on a 32GB GPU (Quadro GV100, Tesla V100-PCIE) with the largest batch size allowed by the memory (8–16 per batch for SGMs, 64 per batch for TransUnet).

### Evaluation metrics

For evaluation, brain segmentation was performed using Freesurfer’s Destrieux parcellation (Destrieux et al., 2009) and related small regions belonging to the same overarching larger region were grouped together (e.g., all sub-regions within frontal cortex were grouped into frontal cortex, more detail in Supplementary Table 1). We performed measurements in 10 region-of-interests (ROI): frontal cortex (FC), temporal cortex (TC), parietal cortex (PC), occipital cortex (OC), insular cortex (IC), cerebral white matter (CWM), deep gray matter including caudate, putamen, and globus pallidus (DGM), combined hippocampus and amygdala (HipAmy), cerebrospinal fluid (CSF), and cerebellum. SUVR was calculated using the cerebellum as the reference region. The mean absolute SUVR difference ( ΔSUVR Mean) and SUVR standard deviations ( ΔSUVR STD) between the acquired and synthetic PET was computed on a voxel-wise basis across the whole brain and across all test subjects. This evaluates the accuracy and consistency of model performance on a voxel level.

Given the diagnostic importance of hemispheric metabolic differences in epilepsy (Lu et al., 2013), we calculated a clinically relevant metric termed the Congruence Index (CI) to assess the agreement of SUVR asymmetry between the synthesized and acquired PET images. We first construct an Asymmetry Index (AI) (Soma et al., 2012; Didelot et al., 2010; Lucas et al., 2024) for each paired left–right ROI using Equation 14, where the difference in SUVR from the left and right hemisphere is divided by the sum. As such, a positive AI indicates right-sided hypometabolism while a negative AI indicates left-sided hypometabolism.


$${\mathrm{AI}}_{\mathrm{ROI}}(\mathrm{PET})=\frac{{\text{SUVR}}_{\text{left}\;\mathrm{ROI}}(\mathrm{PET})-{\text{SUVR}}_{\text{right}\;\mathrm{ROI}}(\mathrm{PET})}{{\text{SUVR}}_{\text{left}\;\mathrm{ROI}}(\mathrm{PET})+{\text{SUVR}}_{\text{right}\;\mathrm{ROI}}(\mathrm{PET})}$$ (14)

Next, we constructed a Congruence Index using formulation in Equation 15 to obtain the proportion of ROIs matching the asymmetry present in the acquired PET. We identified 8 areas with laterality: FC, TC, OC, IC, PC, CWM, DGM, and HipAmy. We averaged the index across the 10 test subjects. The formulation for CI calculation is summarized in Equation 15. In essence, CI measures the degree of agreement between hemispheric uptake asymmetries in the predicted and acquired PET across ROIs, with values ranging from 0 to 1, where 1 indicates perfect agreement.


$$\begin{array}{l} \mathrm{CI}=\frac{1}{\|\text{Subjects}\|*\text{ROIS}}\sum_{\text{Subjects}}\\ \sum_{\text{ROIs}}[[\begin{array}{l} \;{\mathrm{AI}}_{\mathrm{ROI}}({\mathrm{PET}}_{\text{synthetic}})\times {\mathrm{AI}}_{\mathrm{ROI}}\\ ({\mathrm{PET}}_{\text{acquired}})>0\; \end{array}]] \end{array}$$ (15)

Considering the different ROI sizes and the observation that mild asymmetries may be less critical compared with larger asymmetries, we further included the effects of ROI size and absolute differences in a measure termed the Congruence Mean Absolute Error (CMAE) (Equation 16), again averaged across 10 subjects. CMAE is a weighted error for asymmetric agreements between predicted PET and acquired PET, where a smaller value indicates a more accurate synthesis:


$$\begin{array}{l} \text{CMAE}=\frac{1}{\left\|\text{Subjects}\right\|}\sum_{\text{Subjects}}\\ \sum_{\text{ROIs}}\;|{\mathrm{AI}}_{\mathrm{ROI}}({\mathrm{PET}}_{\text{acquired}})\\ -{\mathrm{AI}}_{\mathrm{ROI}}({\mathrm{PET}}_{\text{synthetic}})|*\frac{\mathrm{ROI}}{\text{ROImax}} \end{array}$$ (16)

To analyze slice inconsistencies that can occur using 2D models for 3D image generation, we evaluated high-frequency components across image depth by applying a 1D Fourier transform along the z-axis, followed by averaging the resulting power spectrum across the 2D spatial dimensions and taking the logarithm of the spectral power. We then computed the mean difference in high-frequency power between the acquired and predicted PET images and averaged the differences across test subjects. A negative difference indicates more rapid intensity variation across depth, suggesting increased slice-to-slice inconsistency.

### Statistical analysis

Since SUVR is both subject- and ROI-based, we employed a mixed-effects model to account for both between-group and within-group variance. The mixed-effects framework, as shown in Equation 17, partitions variance into between-subject variance (differences across patients) and within-subject variance (ROI-level). A higher value indicates that the model’s outputs are stable across ROIs within the same subject with low noises (i.e., most of the difference in model performance come from subject variability).


$$\text{Reliability}=\frac{\sigma_{\text{between}-\text{subject}}^{2}}{\sigma_{\text{between}-\text{subject}}^{2}+\sigma_{\text{with}-\text{subject}}^{2}+\sigma_{\text{residual}}^{2}}$$ (17)

Using the variances estimated by this model, we calculated the Intraclass Correlation Coefficient (ICC) to assess the reliability of SUVR values from the synthetic PET images compared to the ground truth. According to Too and Li (2016), ICC values between 0.5 and 0.75 indicate moderate reliability, values between 0.75 and 0.9 indicate good reliability, and higher ICC values reflect greater reliability.

### Bioethics

This study was conducted in accordance with the ethical standards of the institutional research committee and with the Declaration of Helsinki.

## Results

### Patient population

We identified 52 cases (9 non-epilepsy and 43 epilepsy cases). Ten and two epilepsy patients were randomly selected as test and validation cases, respectively. The remainder of the patients were used for model training. The detailed flow chart, the patient demographics with statistical tests can be found in Figure 2 and Table 1 respectively. Based on the clinical reports of the 10 test subjects (Supplementary Table 2), we identified 4 normal cases (no metabolic abnormalities) and 6 abnormal cases with temporal lobe involvement. We first evaluated all 10 cases, and then computed the same metrics separately for the normal and abnormal groups (results provided in the Supplementary Figures 1–3).

**Table 1:** Training and testing data cases.

Characteristics	Training/validation (*n* = 42)	Testing (*n* = 10)	Statistical Test *p*-values
Age	42.2 ± 15.1	34.6 ± 11.1	0.138
Sex	24 female, 18 male	6 female, 4 male	0.532
Radiotracer dose level	2.85 ± 0.65 MBq/kg	3.00 ± 0.73 MBq/kg	0.426

Mann–Whitney U tests were used to assess group differences in age and radiotracer dose, and a Chi-square test was used to assess sex distribution. All *p*-values indicate no significant differences between the training/validation and testing groups.

### Visual quality of synthetic full-dose FDG-PET

**Figure 3:**
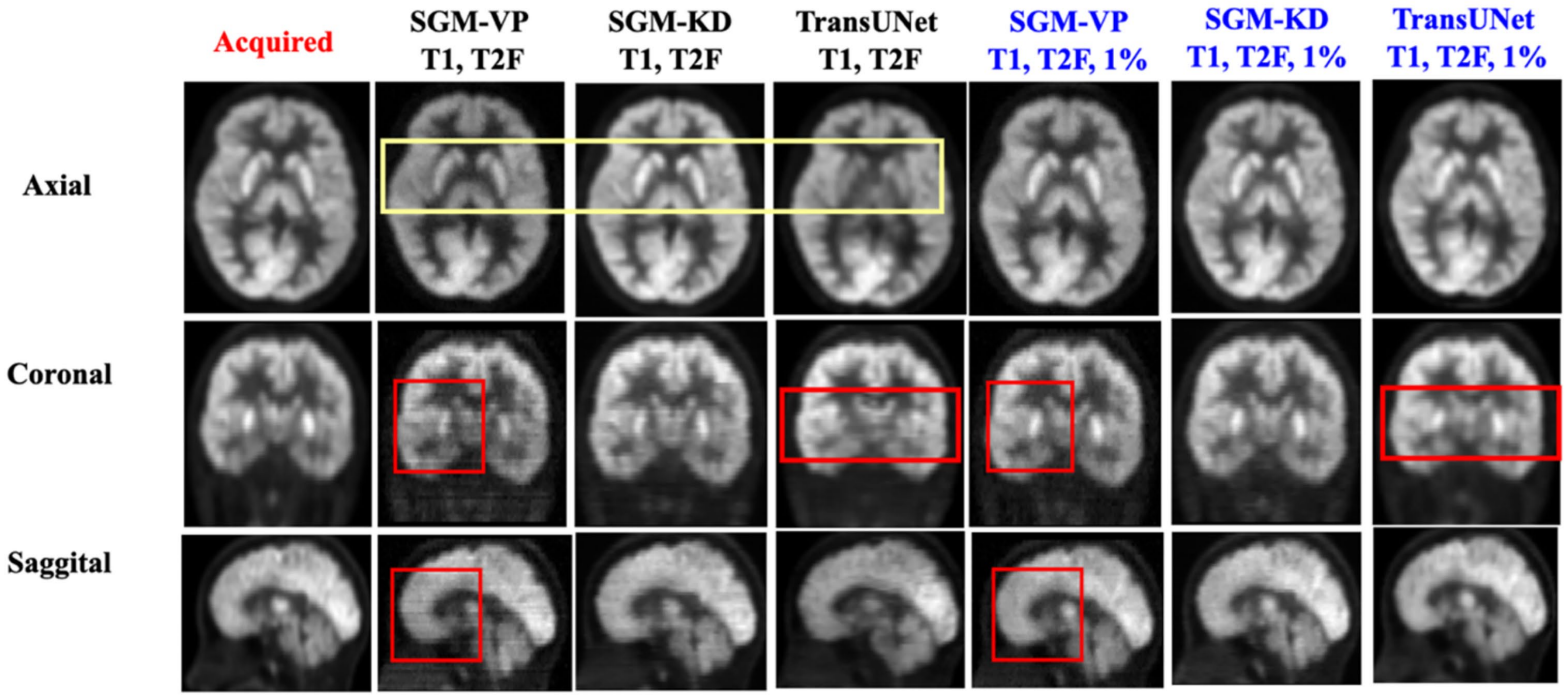
Slice visualization for a test subject in all 3 views for different models and different inputs. SGM-VP and SGM-KD are score-based diffusion models with variations in the noise scheduling. The yellow box compares the anatomical details generated by 3 models in zero-dose task, which demonstrates less accurate detail for the TransUnet model, particularly in the region of the basal ganglia. The red box indicates the effect of the 1% PET input in improving the slice consistency in coronal and sagittal view and anatomical details (moving from black columns to blue columns). This improvement is the most significant for TransUnet, emphasizing the importance of ultralow-dose inputs.

**Figure 4:**
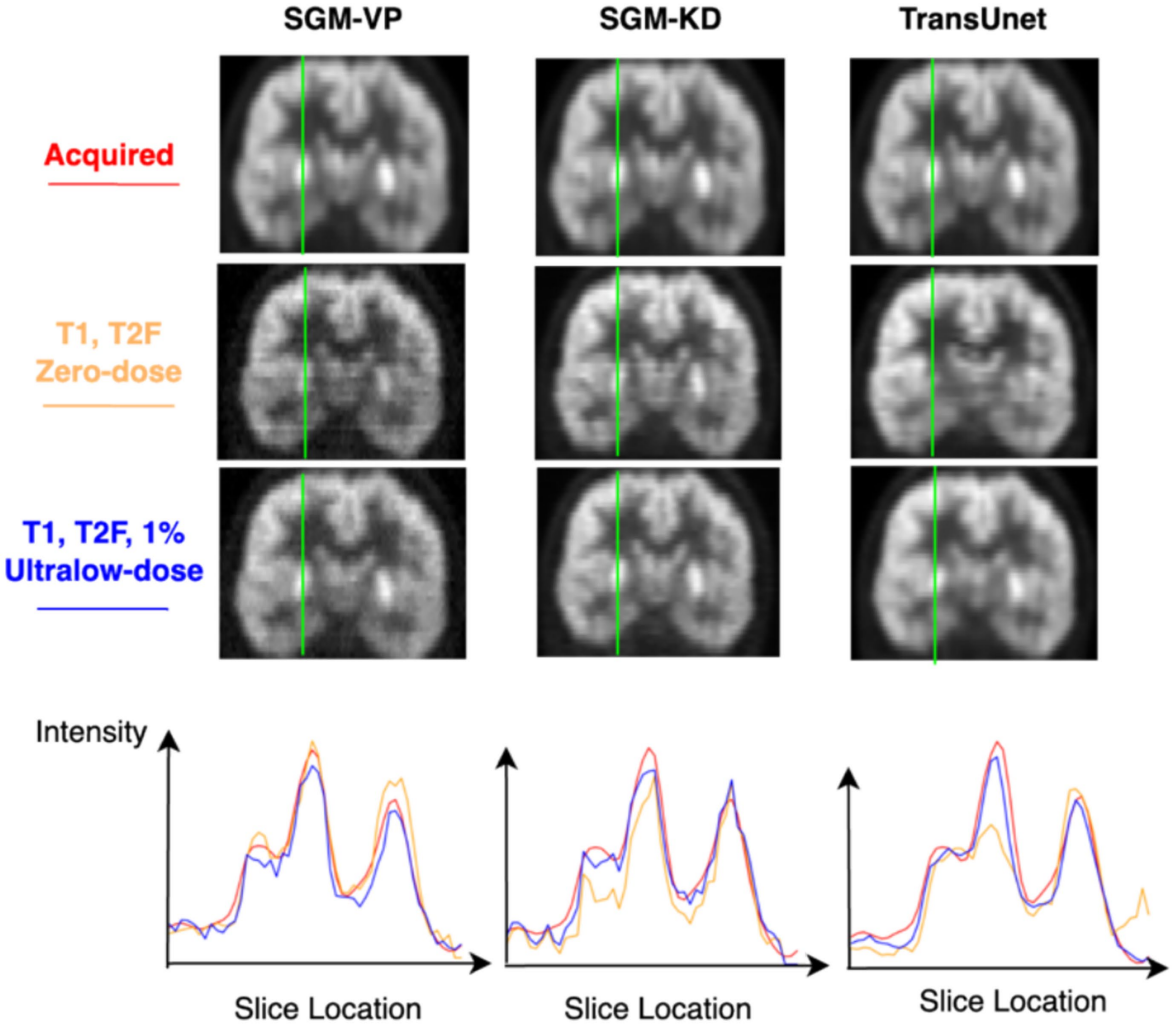
Interslice consistency evaluation between zero-dose and ultralow-dose tasks using the line profile from 1 coronal slice in each PET (intensity values taken along the green lines). On the bottom graphs, the red curve represents the acquired PET, the orange curve represents the zero-dose task, and the blue curve represents the ultralow-dose task. The blue curve is closer to the red curve and has less high-frequency components, indicating improved slice intensity consistency for the ultralow-dose task.

**Figure 5:**
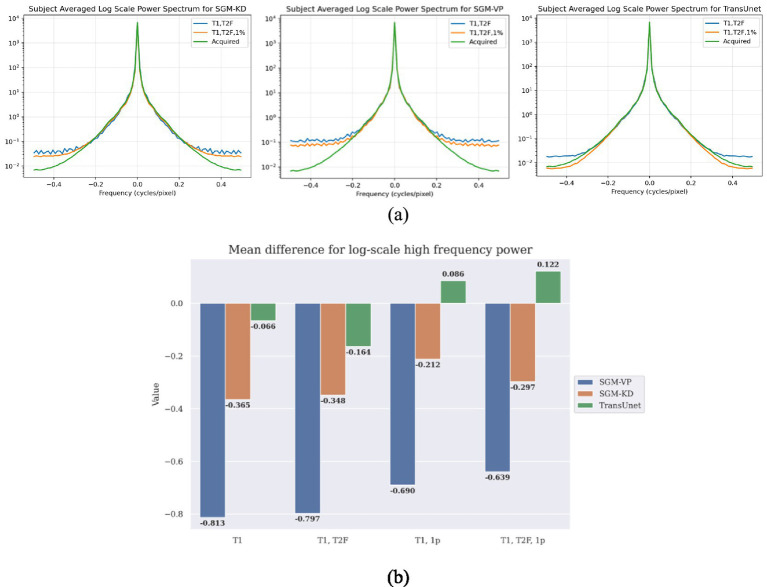
**(a)** The Fourier transform power spectrum in log-scale averaged across 10 test subjects. There is more power in higher frequencies in SGM-KD and SGM-VP, showing the slice inconsistency issue observed earlier. The inconsistency is reduced when PET 1% dose images are included (orange), particularly for the TransUnet. **(b)** Difference in log-scale high-frequency power between acquired and predicted PET images for different model and input combinations. Negative values indicate greater high-frequency content in the predicted PET, corresponding to increased slice-to-slice inconsistency. The inclusion of 1% PET input reduced high-frequency components, indicating improved slice consistency. TransUnet exhibited minimal slice inconsistency in both tasks.

For the zero-dose task using only MRI sequences as inputs, the 2 SGM models outperformed the TransUnet, as shown by the more accurate synthesis in the region highlighted by the yellow boxes in Figure 3. The TransUnet (CNN style network) tended to generate oversmoothed images with fewer details, consistent with the observation made in (Lyu and Wang, 2022), whereas score-based models emphasize the edges and the contrast between regions with low and high metabolism. For the ultralow-dose task that included 1% dose PET images as input, the two SGM models exhibited reduced slice-to-slice inconsistencies in coronal and sagittal views, improving the quality of coronal and sagittal views (Figure 3, and more examples in Supplementary materials). This is also seen in Figure 4, which provides a closer examination of the slice inconsistency improvement moving from zero-dose to ultralow-dose task. The ultralow-dose line profiles exhibit less jittering compared with the zero-dose profiles, reflecting smoother intensity transitions between slices that more closely resemble the acquired PET. This is further quantified by applying a Fourier transform along the z direction (cranial-caudad) to quantify slice-to-slice inconsistencies. As shown in Figure 5a, we averaged the log-scale power spectra across 10 subjects for the acquired, zero-dose, and ultralow-dose PET images. Increased high-frequency fluctuations correspond to more rapid intensity changes across depth, indicative of greater slice inconsistency. These high-frequency components were more pronounced in the zero-dose task (blue curve) compared to the ultralow-dose task (orange curve). TransUnet exhibited reduced slice inconsistency, as reflected by the smoother high-frequency tails shown in the right panel.

Notably, the TransUnet shows the least slice inconsistency in the zero-dose task, but requires 1% PET input to generate sufficient anatomical detail. This limitation may stem from its reduced complexity, which could hinder performance with limited training data.

To further quantify this effect, we computed the average high-frequency power difference (> 0.2 cycles/pixel) using ${\text{Power}}_{\text{acquired}}-{\text{Power}}_{\text{predicted}}$ , summarized in Figure 5b and Supplementary Table 3. We observed larger high-frequency discrepancies in the zero-dose task when using diffusion-based models.

**Figure 6:**
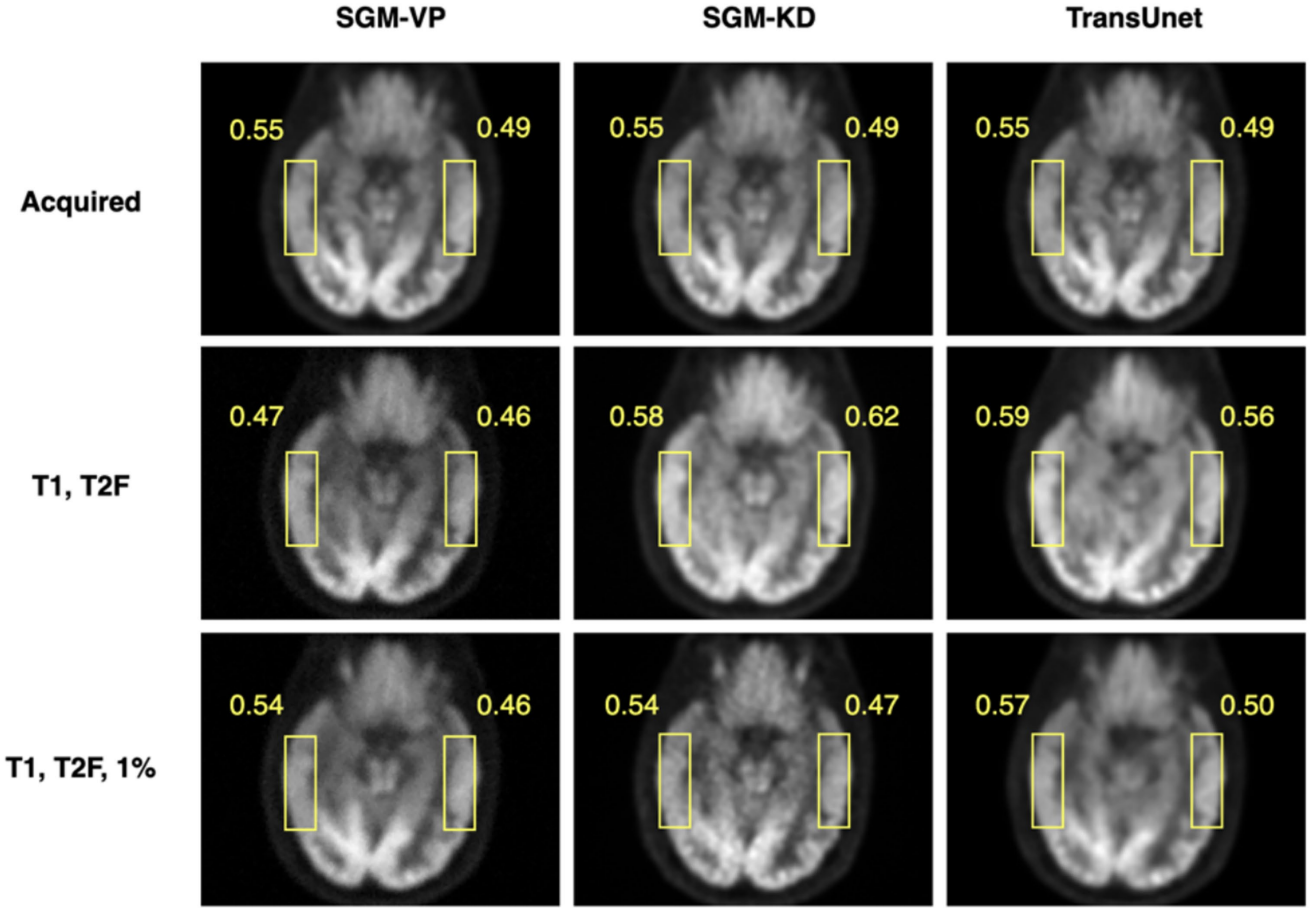
Axial slice for one case with asymmetrically lower metabolism in the left temporal lobe based on the clinical report. We computed the mean SUVR in the left and right yellow boxes with values shown on top. We observe better agreement with the acquired PET for ultralow-dose task compared to zero-dose task.

Lastly, we examined the quality of the synthetic PET for a test case with left-sided temporal lobe hypometabolism based on the clinical reports. As shown in Figure 6, the left–right asymmetries are much easier to identify on the ultralow-dose synthesis as compared to the zero-dose synthesis. This is true for all three models.

### Quantitative performance

Next, we evaluated the quantitative aspects of the synthesized images for zero-dose task (Table 2). The SGM-VP model demonstrated the best performance for the asymmetry metrics CI and CMAE, indicating that SGM-VP is more accurate for larger proportion of the test subjects and ROIs. The ICC for all models is around 0.75 or greater, suggesting that they all have good reliability. While ICCs are all relatively comparable, the SGM-KD model using T1w and T2-FLAIR as inputs had the highest ICC (0.84).

**Table 2:** Performance metrics for the zero-dose task with different MR inputs.

Model type	Inputs	SUVR ICC	SUVR ICC 95% CI	CMAE	CMAE 95% CI	Congruence index	Congruence index 95% CI
SGM-VP	T1w	0.81	0.65, 0.91	**0.61**	0.23, 0.99	**0.85**	0.73, 0.97
SGM-VP	T1w, T2F	0.80	0.63, 0.90	1.11	0.67, 1.56	0.76	0.70, 0.83
SGM-KD	T1w	0.82	0.67, 0.91	1.10	0.71, 1.49	0.71	0.60, 0.82
SGM-KD	T1w, T2F	**0.84**	0.70, 0.92	0.73	0.30 1.15	0.76	0.66, 0.87
TransUnet	T1w	0.80	0.63, 0.90	1.01	0.48, 1.54	0.74	0.62, 0.86
TransUnet	T1w, T2F	0.74	0.55, 0.87	1.07	0.45, 1.70	0.61	0.51, 0.71

The best performance for each metric is shown in bold. The 95% confidence interval for Congruence Index and Congruence Mean Absolute Error was calculated using the t-distribution. CMAE is scaled by 1,000 for better visualization, and a lower value indicates better performance.

**Figure 7:**
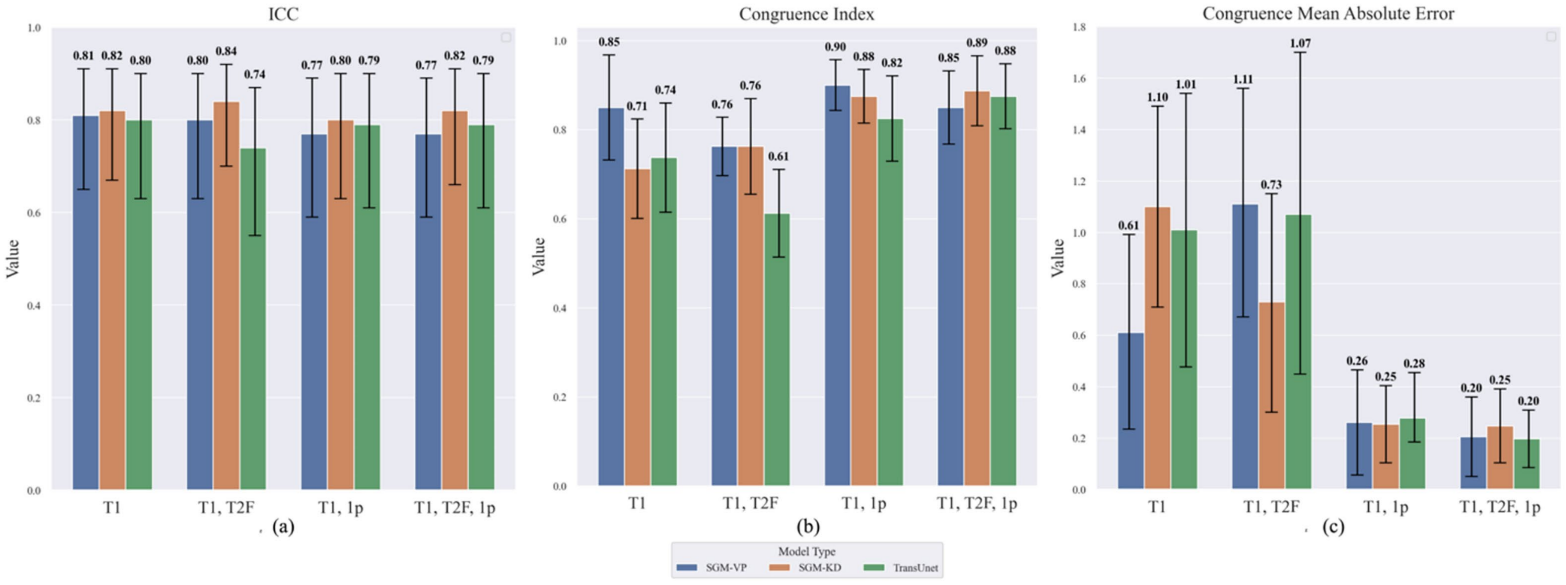
Visualization of **(a)** ICC, **(b)** CI, and **(c)** CMAE for the different models and image input conditions. **(a)** No significant change in ICC was observed between the zero-dose task and ultralow-dose tasks. **(b)** Increases in CI and **(c)** especially decreases in CMAE demonstrate significant improvement for models that include 1% dose PET as an input. Please note that higher CI and lower CMAE indicates better performance.

When PET 1% data is included as input, there is improved performance for the asymmetry indices for all model types (Table 3). Specifically, the best performing CI increases to a high of 0.90 while the best CMAE is 0.197, approximately 3 times lower than that for the best zero-dose model. TransUnet experiences the largest improvement compared to SGMs and is now capable of capturing smaller anatomical details. ICC values were largely unchanged, still exceeding the 0.75 threshold indicative of good reliability, suggesting that ICC is less dependent on the presence of ultralow-dose inputs. Figure 7 presents these findings in a summary chart that allows clear visualization of the improvement of the CI and CMAE metrics for all models when the 1% PET image is included as an input.

**Table 3:** Performance metrics for the ultralow-dose PET + MRI inputs.

Model type	Inputs	SUVR ICC	SUVR ICC 95% CI	CMAE	CMAE 95% CI	Congruence index	Congruence index 95% CI
SGM-VP	T1w, 1%	0.77	0.59, 0.89	0.261	0.056, 0.47	**0.90**	0.84, 0.96
SGM-VP	T1w, T2F, 1%	0.77	0.59, 0.89	0.205	0.050, 0.36	0.85	0.77, 0.93
SGM-KD	T1w, 1%	0.80	0.63, 0.90	0.254	0.10, 0.40	0.88	0.82, 0.94
SGM-KD	T1w, T2F, 1%	**0.82**	0.66, 0.91	0.247	0.10, 0.39	0.89	0.81, 0.97
TransUnet	Tw1, 1%	0.79	0.61, 0.90	0.278	0.18, 0.45	0.83	0.73, 0.92
TransUnet	T1w, T2F, 1%	0.79	0.61, 0.90	**0.197**	0.086, 0.31	0.88	0.80, 0.95

The best performance for each metric is in bold. The 95% confidence interval for Congruence Index and Congruence Mean Absolute Error was calculated using the t-distribution. CMAE is scaled by 1,000 for better visualization, and a lower value indicates better performance.

**Figure 8:**
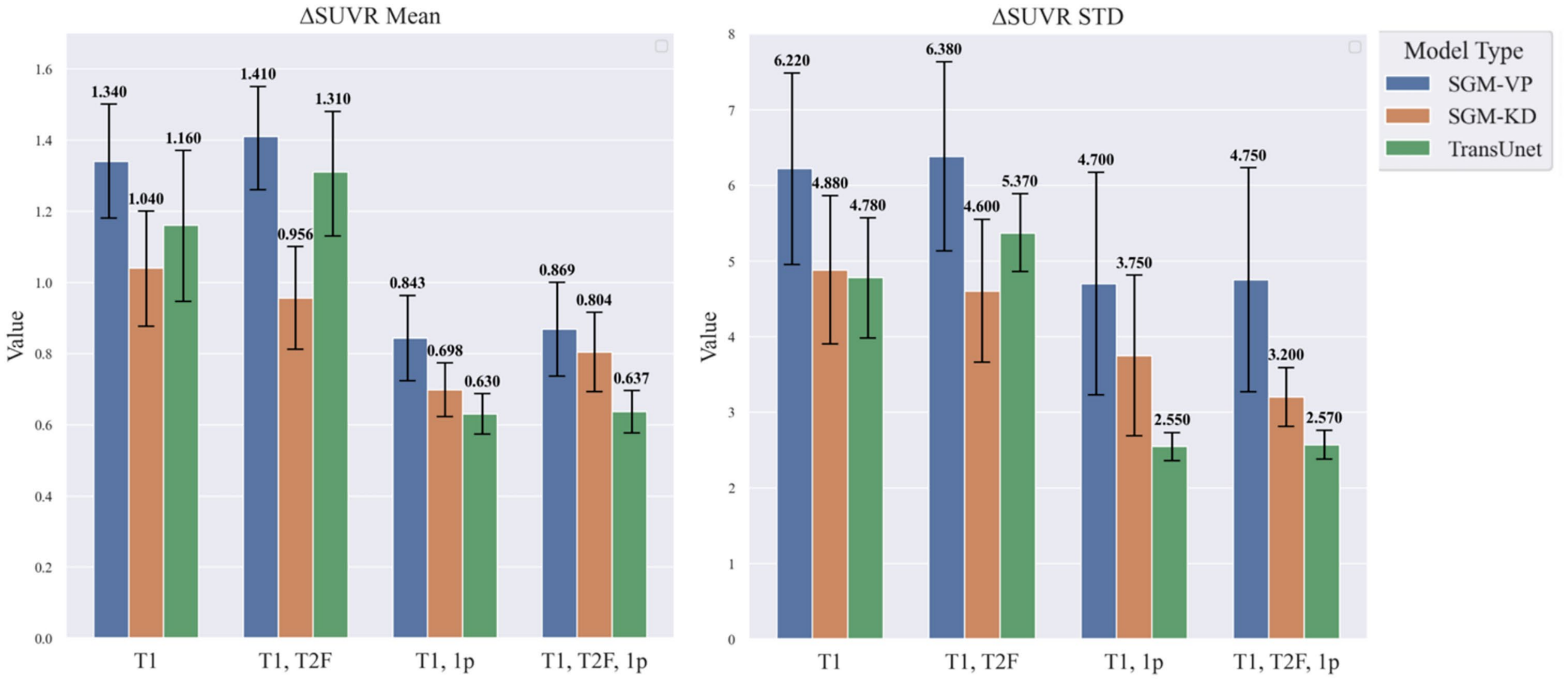
Visualization of Δ SUVR ean and STD for different models and image input conditions. There is decreased Δ SUVR mean and standard deviation from zero-dose to ultralow-dose, indicating overall higher voxel-wise accuracy in synthesis.

The performances on mean voxel-wise Δ SUVR and mean Δ SUVR standard deviation are shown in Table 4 and Figure 8, where the top half represents the zero-dose task and the lower half the ultralow-dose task. For the zero-dose task, SGM-KD showed both the lowest Δ SUVR Mean and STD, meaning that although SGM-KD is not the best for asymmetry measures, it has a more balanced performance across the volume. For the ultralow-dose task, TransUnet has the lowest Δ SUVR Mean and STD. We notice that although SGM-VP performs better for asymmetry measures, SGM-KD outperforms in the Δ SUVR metrics. A possible explanation is the limited dataset size, which may introduce differences that would not otherwise occur. Additionally, the architecture of SGM-KD might be more effective at synthesizing pixels with average intensities, rather than those representing hypo- or hyper-metabolized regions at the distribution’s extremes. However, these performance differences may change when more data becomes available.

**Table 4:** Performances for ΔSUVR Mean and ΔSUVR STD.

Model type	Inputs	ΔSUVR Mean (x100)	ΔSUVR Mean CI (x100)	ΔSUVR STD (x100)	ΔSUVR STD CI (x100)
Zero-dose inputs					
SGM-VP	T1w	1.34	1.18, 1.50	6.22	4.95, 7.48
SGM-VP	T1w, T2F	1.41	1.26, 1.55	6.38	5.13, 7.63
SGM-KD	T1w	1.04	0.88, 1.20	4.88	3.90, 5.86
SGM-KD	T1w, T2F	**0.96**	0.81, 1.10	**4.60**	3.66, 5.55
TransUnet	T1w	1.16	0.94, 1.37	4.78	3.98, 5.57
TransUnet	T1w, T2F	1.31	1.13, 1.48	5.37	4.86, 5.89
Ultralow-dose inputs					
SGM-VP	T1w, 1%	0.84	0.72, 0.96	4.70	3.23, 6.17
SGM-VP	T1w, T2F, 1%	0.87	0.74, 1.00	4.75	3.27, 6.23
SGM-KD	T1w, 1%	0.70	0.62, 0.77	3.75	2.69, 4.81
SGM-KD	T1w, T2F, 1%	0.80	0.69, 0.92	3.20	2.81, 3.59
TransUnet	T1w, 1%	**0.63**	0.57, 0.69	**2.55**	2.36, 2.73
TransUnet	T1w, T2F, 1%	0.64	0.58, 0.70	2.57	2.38, 2.76

All values are upscaled by 100 for better visualization purposes. The best performances in both tasks are in bold. For zero-dose task, the best performance is SGM-KD using T1w and T2F, while for ultralow-dose task, the best performance is TransUnet using T1w and PET 1%.

The CI, CMAE and Δ SUVR metrics for normal cases and abnormal cases are presented in Supplementary Figures 1–3. We observed a consistent trend across both groups, where the inclusion of PET 1% led to improved performances. Performance overall was similar between the groups.

### Performance in temporal lobe cortex

**Figure 9:**
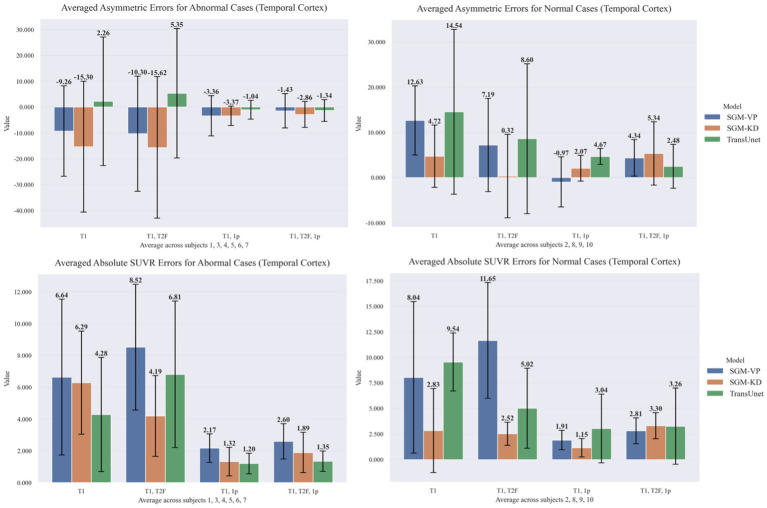
Visualizations of temporal lobe cortex specific asymmetric errors and SUVR errors for the normal cases and abnormal cases.

Due to the important role of the temporal lobe in epilepsy diagnosis using PET imaging, we evaluated performance within this specific ROI for normal cases (n = 4) and abnormal cases (n = 6). As shown in Figure 9, the inclusion of 1% PET input improved asymmetry and SUVR performance across nearly all model and input combinations in both groups.

Lastly, TransUnet demonstrates significantly faster sampling speeds compared to diffusion models, with inference for the entire 89 slice volume of under 40 s, whereas SGM-KD requires approximately 400 s and SGM-VP takes around 2,420 s using 8-batch inference.

## Discussion

In this study, we comprehensively examined the performance of both TransUnet and score-based generative models to synthesize full dose FDG brain PET imaging in epilepsy patients using inputs that include either only MRI or MRI plus ultra-low 1% dose PET images. We incorporate more medical-imaging relevant metrics and quantifications in addition to more traditional metrics for natural images. We found that score-based diffusion models demonstrate stable performance, are less dependent on ultralow-dose PET inputs, and outperform a TransUnet model for zero-dose tasks. However, they suffer from slower sampling and more noticeable slice-to-slice intensity inconsistencies, which are suboptimal for volumetric evaluation. All the models demonstrated ICC values consistent with at least good reliability. Recognizing the complexity of real-world diagnosis that integrates MRI, EEG, and clinical information, we focused our evaluation on clinically meaningful metabolic asymmetries to better address the challenges of quantitative measurement in medical imaging.

Previously, Xiang et al. (2017) developed a CNN-based patch model for low-dose (25%) PET denoising that incorporated T1w MRI information. Their approach used low-dose PET data acquired during a 3 min short scan extracted from a 12 min standard-dose tracer injection, which differs from the event under-sampling strategy we employed during list-mode reconstruction. Another prior study examining only MR-to-PET image modality synthesis integrated transformer, generator, and discriminator components into a U-Net architecture to synthesize FDG-PET images from multi-modal MR inputs, including T1w, T1c, T2 FLAIR, and ASL in brain tumor patients (Ouyang et al., 2024). While this approach demonstrated strong performance based on metabolic metrics such as SUVR, it relied on the availability of diverse contrast images, which may not always align with clinical acquisition protocols. In contrast, the current study utilizes fewer and more basic MRI inputs, improving the practicality and generalizability of the method across diverse datasets. Additionally, the availability of list-mode data provides higher flexibility to simulate different dose levels, facilitating explorations into dose optimization.

Due to the scarcity of publicly available PET datasets, particularly for epilepsy cases, we first examined prior work on PET reconstruction and synthesis in dementia studies. The availability of large ADNI datasets has enabled the development of multiple GAN-based 3D image translation approaches (Li et al., 2014; Hinge et al., 2022; Sikka et al., 2018). For instance, Li et al. (2014) applied patch-based 3D CNNs for MRI-to-PET translation using 19.9 million patches from ADNI. Hinge et al. (2022) employed a Pix2Pix conditional GAN, while Sikka et al. (2018) utilized a 3D U-Net on full MR volumes to capture global context, improving Alzheimer’s classification using synthetic PET. Similarly, Pan et al. (2018) used a cycle-consistent GAN to impute missing PET data, integrating it with MRI for AD diagnosis. With a focus on clinical applications, Choi et al. (2019) trained a variational autoencoder (VAE) on normal brain PET to assist in AD and MCI diagnosis. For amyloid PET reconstruction, Ouyang et al. (2019) proposed a 2.5D GAN model with task-specific perceptual loss, leveraging a pre-trained amyloid status classifier to enhance pathological feature matching. Additionally, Doering et al. (2025) used combined CNN and patient feature integration for MRI-tauPET translation. However, despite these advancements, the exploration of diffusion architectures remains significantly underdeveloped, likely due to their high computational demands. Diffusion models hold great promise for real-world applications in image processing and acquisition. For example, a denoising diffusion null-space model was trained on high-quality PET images from a high-end scanner and integrated into a conventional PET system to enhance lower-quality scans (Zhang et al., 2024). This highlights the realistic potential of diffusion-based methods to improve PET imaging, warranting further investigation into their feasibility and impact.

Specifically in the field of epilepsy, Yaakub et al. synthesized what they term “pseudo-normal” PET images from T1w MRI using a GAN trained on healthy subjects. This model was then compared with an acquired FDG brain PET using a z-score metric, with areas of discrepancy highlighting potential seizure-affected regions, demonstrating about 92% accuracy for localization of the epileptic zone (Yaakub et al., 2019). Unlike their emphasis on comparing a full-dose FDG brain PET image to a synthesized pseudonormal scan for the purposes of diagnostic insight, our work prioritizes reducing radiation exposure while emphasizing accurate clinical indicators such as hemisphere asymmetries.

There are several important considerations when choosing a generative model to synthesize full-dose FDG PET. TransUnet allows the fastest inference (<40s per volume) but needs larger training data or more input information. Thus, it is the most suitable for larger training sets containing both MRI and low-dose PET images. Of the two score-based diffusion models, SGM-KD has shorter sampling time than SGM-VP due to its use of step discretization. Specifically, we can limit the number of sampling steps to 50–150 steps (<37 s per 8-slice inference batch, ~400 s per volume) for a satisfactory result, compared to 200–300 steps that are required for the SGM-VP (<220 s per 8-slice inference batch, ~2,420 s per volume). The importance of sampling efficiency depends on the clinical use case. In non-emergency settings, accuracy may take precedence over efficiency. Ongoing work aims to reduce the sampling time of SGMs, including approaches such as consistency models (Song et al., 2023) and DPM-based samplers (Lu et al., 2022).

The best model also depends on the evaluation metric of interest and the available inputs. SGM-KD, while having the lowest mean ΔSUVR and the highest ICC for zero-dose task, may not be as accurate as SGM-VP in terms of capturing hemisphere asymmetries. For the ultralow-dose task, TransUnet outperformed on these congruency-based symmetry measures, ΔSUVR error, and sampling efficiency, but has slightly lower performance based on ICC, though still in the range indicating good consistency. This emphasizes that the choice of a model may depend on its desired application, adding a level of complexity to the selection process.

It is important to note that the limited training data and variations in model architecture and training time may contribute to differences in model performance. These may diminish with access to larger datasets, as models typically converge to better performance when trained on more comprehensive data. It is important to note the limitation of this study using only single-center data, which reduces harmonization confounders but may oversimplify the reconstruction task relative to multi-center scenarios. As a proof-of-principle study, demonstrating that data from one scanner can be successfully reproduced suggests that generalization to multi-center data may be achievable with sufficiently diverse training cohorts. In future work, multi-center variability could be addressed either by incorporating real multi-site data or by augmenting with synthetically generated scanner-style variations learned from existing data. The ultralow-dose PET was simulated from full-dose injections, which may not fully represent PET images acquired with lower dose levels, though prior studies with true ultralow-dose have suggested good concordance with similar simulations (Chen et al., 2021). Additionally, our analysis was restricted to a subset of deep learning models commonly applied in medical imaging, leaving room for broader exploration.

Finally, a reader study to evaluate the clinical utility of the synthetic PET images remains an important area for future investigation and also to see the extent to which slice inconsistencies may be an issue. Future studies using larger numbers of patients with a PET reader study format would be required to evaluate higher level questions such as its ability to accurately depict FDG changes related to focal cortical dysplasia or remote hypometabolism valuable for epilepsy.

## Conclusion

In an epilepsy patient population, we demonstrated the ability to synthesize full-dose FDG-PET brain images from MRI inputs using deep learning diffusion models. The potential to improve performance by including ultralow 1% dose FDG-PET images creates high clinical value and real-life applicability. Future work needs to validate the accuracy of low-dose simulation through real 1% tracer injection. Will focus on refining slice consistency through averaging techniques (Choo et al., 2024), exploring 3D full volume and patch-based approaches, improving ultralow-dose reconstruction quality, and assessing whether evaluation metrics such as Congruence Index (CI) correlates with findings from reader studies. Additionally, we plan to explore multi-task brain MRI-to-PET translation, including different tracers (amyloid, tau) and different disease conditions (tumor, dementia, etc.). Incorporating clinical reports as additional conditioning inputs may further enhance model performance. Overall, this study highlights the potential of generative AI approaches to significantly reduce brain FDG-PET dose levels, offering substantial clinical benefits for patients.

## Data Availability

The imaging data used in this study are not publicly available due to patient privacy concerns and institutional data use agreements. Data may be made available upon reasonable request and subject to approval by the corresponding author’s Institutional Review Board (IRB) and relevant data sharing agreements. Requests to access these datasets should be directed to GZ, gregz@stanford.edu.

## References

[ref1] Armanious K. Jiang C. Fischer M. Kustner T. Hepp T. Nikolaou K. . (2020). MedGAN: medical image translation using GANs. Comput. Med. Imaging Graph. 79:101684. doi: 10.1016/j.compmedimag.2019.101684 31812132

[ref2] Cendes F. Theodore W. H. Brinkmann B. H. Sulc V. Cascino G. D. (2016). “Chapter 51 - neuroimaging of epilepsy,” in Neuroimaging Part II. vol. 136 of Handbook of Clinical Neurology, eds. Masdeu J. C. Gonz’alez R. G. (Amsterdam, Netherlands: Elsevier), 985–1014. 10.1016/B978-0-444-53486-6.00051-X PMC5256664 27430454

[ref3] Chen K. T. Gong E. de Carvalho Macruz F. B. Xu J. Boumis A. Khalighi M. . (2020). Ultra–low-dose 18F-florbetaben amyloid PET imaging using deep learning with multi-contrast MRI inputs. Radiology 296:E195. doi: 10.1148/radiol.2020202527, 32804601 PMC8906337

[ref4] Chen J. Lu Y. Yu Q. Luo X. Adeli E. Wang Y. (2021) TransUNet: transformers make strong encoders for medical image segmentation. Available online at: https://arxiv.org/abs/2102.04306 (Accessed October 01, 2025).

[ref5] Chen K. T. Toueg T. N. Koran M. E. I. Davidzon G. Zeineh M. Holley D. . (2021). True ultra-low-dose amyloid PET/MRI enhanced with deep learning for clinical interpretation. Eur. J. Nucl. Med. Mol. Imaging 48, 2416–2425. doi: 10.1007/s00259-020-05151-9, 33416955 PMC8891344

[ref6] Choi H. Ha S. Kang H. Lee H. Lee D. S. A. D. N. I. (2019). Deep learning only by normal brain PET identify unheralded brain anomalies. EBioMedicine 43, 447–453. doi: 10.1016/j.ebiom.2019.04.022, 31003928 PMC6557913

[ref7] Choo K. Jun Y. Yun M. Hwang S. J. (2024). “Slice-consistent 3D volumetric brain CT-to-MRI translation with 2D Brownian bridge diffusion model,” in Medical Image Computing and Computer Assisted Intervention – MICCAI 2024, eds. Linguraru M. G. Dou Q. Feragen A. Giannarou S. Glocker B. Lekadir K. . (Cham: Springer Nature Switzerland), 657–667.

[ref8] Deidda D. Karakatsanis N. Robson P. M. Efthimiou N. Fayad Z. A. Aykroyd R. G. . (2019). Effect of PET-MR inconsistency in the kernel image reconstruction method. IEEE Trans. Radiat. Plasma Med. Sci. 3, 400–409. doi: 10.1109/TRPMS.2018.2884176, 33134651 PMC7596768

[ref9] Destrieux C. Fischl B. Dale A. Halgren E. (2009). A sulcal depth-based anatomical parcellation of the cerebral cortex. NeuroImage 47, S39–S41. doi: 10.1016/S1053-8119(09)71561-7

[ref10] Didelot A. Maugui’ere F. Redout’e J. Bouvard S. Lothe A. Reilhac A. . (2010). Voxel-based analysis of asymmetry index maps increases the specificity of 18F-MPPF PET abnormalities for localizing the epileptogenic zone in temporal lobe epilepsies. J. Nucl. Med. 51, 1732–1739. doi: 10.2967/jnumed.109.070938 21051649

[ref11] Doering E. Hoenig M. Bischof G. van Eimeren T. Drzezga A. (2025). MRI-to-PET translation with patient feature integration for the diagnosis of Alzheimer’s disease. J. Nucl. Med. 66:251062.

[ref12] Fu Y. Dong S. Huang Y. Niu M. Ni C. Yu L. . (2024). MPGAN: Multi Pareto Generative Adversarial Network for the denoising and quantitative analysis of low-dose PET images of human brain. Med Image Anal 98:103306. doi: 10.1016/j.media.2024.103306 39163786

[ref13] Gallach M. Lette M. M. Abdel-Wahab M. Giammarile F. Pellet O. Paez D. (2020). Addressing global inequities in positron emission tomography-computed tomography (PET-CT) for cancer management: a statistical model to guide strategic planning. Med. Sci. Monit. 26:e926544. doi: 10.12659/MSM.926544, 32848125 PMC7476356

[ref14] Gong K. Johnson K. El Fakhri G. Li Q. Pan T. (2024). PET image denoising based on denoising diffusion probabilistic model. Eur. J. Nucl. Med. Mol. Imaging 51, 358–368. doi: 10.1007/s00259-023-06417-8 37787849, 37787849 PMC10958486

[ref15] Hinge C. Henriksen O. M. Lindberg U. Hasselbalch S. G. Højgaard L. Law I. . (2022). A zero-dose synthetic baseline for the personalized analysis of [18F]FDG-PET: application in Alzheimer’s disease. Front. Neurosci. 16:1053783. doi: 10.3389/fnins.2022.1053783, 36532287 PMC9749397

[ref16] Huang B. Law M. W. M. Khong P. L. (2009). Whole-body PET/CT scanning: estimation of radiation dose and Cancer risk. Radiology 251, 166–174. 19251940. doi: 10.1148/radiol.2511081300, 19251940

[ref17] Jiang H. Imran M. Ma L. Zhang T. Zhou Y. Liang M. (2024) Fast-DDPM: fast denoising diffusion probabilistic models for medical image-to-image generation. Available online at: https://arxiv.org/abs/2405.14802 (Accessed June 01, 2024). 10.1109/JBHI.2025.3565183 40293895

[ref18] Karras T. Aittala M. Aila T. Laine S. (2022) Elucidating the design space of diffusion-based generative models. Available online at: https://arxiv.org/abs/2206.00364 (Accessed October 01, 2024).

[ref19] Kikuchi K. Togao O. Yamashita K. Momosaka D. Nakayama T. Kitamura Y. . (2021). Diagnostic accuracy for the epileptogenic zone detection in focal epilepsy could be higher in FDG-PET/MRI than in FDG-PET/CT. Eur. Radiol. 31, 2915–2922. doi: 10.1007/s00330-020-07389-1, 33063184 PMC8043950

[ref20] Li H. Badawi R. D. Cherry S. R. Fontaine K. He L. Henry S. . (2024). Performance characteristics of the NeuroEXPLORER, a next-generation human brain PET/CT imager. J. Nucl. Med. 65, 881–890. doi: 10.2967/jnumed.124.267767 PMC11294061 38871391

[ref21] Li R. Zhang W. Suk H. I. Wang L. Li J. Shen D. . (2014). Deep learning based imaging data completion for improved brain disease diagnosis. Med Image Comput Comput Assist Interv. 17, 305–312. doi: 10.1007/978-3-319-10443-0_39, 25320813 PMC4464771

[ref22] Lu J. Li W. He H. Feng F. Jin Z. Wu L. (2013). Altered hemispheric symmetry found in left-sided mesial temporal lobe epilepsy with hippocampal sclerosis (MTLE/HS) but not found in right-sided MTLE/HS. Magn. Reson. Imaging 31, 53–59. doi: 10.1016/j.mri.2012.06.030, 22925605

[ref23] Lu C. Zhou Y. Bao F. Chen J. Li C. Zhu J. (2022) DPM-solver: a fast ODE solver for diffusion probabilistic model sampling in around 10 steps. Available online at: https://arxiv.org/abs/2206.00927 (Accessed June 01, 2024).

[ref24] Lucas A. Vadali C. Mouchtaris S. Arnold T. C. Gugger J. J. Kulick-Soper C. . (2024). Enhancing the diagnostic utility of ASL imaging in temporal lobe epilepsy through FlowGAN: an ASL to PET image translation framework. medRxiv. doi: 10.1101/2024.05.28.24308027

[ref25] Lyu Q. Wang G. (2022) Conversion between CT and MRI images using diffusion and score-matching models. Available online at: https://arxiv.org/abs/2209.12104 (Accessed July 01, 2024).

[ref26] Ouyang J. Chen K. T. Duarte Armindo R. Davidzon G. A. Hawk K. E. Moradi F. . (2024). Predicting FDG-PET images from multi-contrast MRI using deep learning in patients with brain neoplasms. J. Magn. Reson. Imaging 59, 1010–1020. doi: 10.1002/jmri.28837, 37259967 PMC10689577

[ref27] Ouyang J. Chen K. T. Gong E. Pauly J. Zaharchuk G. (2019). Ultra-low dose PET reconstruction using generative adversarial network with feature mapping and task-specific perceptual loss. Med. Phys. 46, 3555–3564. doi: 10.1002/mp.13626, 31131901 PMC6692211

[ref28] Pan Y. Liu M. Lian C. Zhou T. Xia Y. Shen D. (2018). “Synthesizing missing PET from MRI with cycle-consistent generative adversarial networks for Alzheimer’s disease diagnosis,” in Proceedings of MICCAI. 11072 of Lecture Notes in Computer Science, eds. Frangi A. F. Schnabel J. A. Davatzikos C. Alberola-L’opez C. Fichtinger G. (Cham: Springer), 455–463. 10.1007/978-3-030-00931-1_52 PMC8336606 34355223

[ref29] Ponisio M. R. Zempel J. M. Day B. K. Eisenman L. N. Miller-Thomas M. M. Smyth M. D. . (2021). The role of SPECT and PET in epilepsy. Am. J. Roentgenol. 216, 759–768. doi: 10.2214/AJR.20.23336, 33474983

[ref30] Sikka A. Peri S. V. Bathula D. R. (2018). “MRI to FDG-PET: cross-modal synthesis using 3D U-net for multi-modal Alzheimer’s classification,” in Proceedings of SASHIMI 2018. 11037 of Lecture Notes in Computer Science, eds. Gooya A. Goksel O. Oguz I. Burgos N. (Cham: Springer), 80–89.

[ref31] Singh I. R. Denker A. Barbano R. Kereta Jin B. Thielemans K. Maass P. . (2024). Score-based generative models for PET image reconstruction. Machine Learn. Biomed. Ima. 2, 547–585. doi: 10.59275/j.melba.2024-5d51

[ref32] Soma T. Momose T. Takahashi M. Koyama K. Kawai K. Murase K. . (2012). Usefulness of extent analysis for statistical parametric mapping with asymmetry index using inter-ictal FDG-PET in mesial temporal lobe epilepsy. Ann. Nucl. Med. 26, 319–326. doi: 10.1007/s12149-012-0581-3 22311414

[ref33] Song Y. Dhariwal P. Chen M. Sutskever I. (2023) Consistency models. Available online at: https://arxiv.org/abs/2303.01469 (Accessed October 01, 2025).

[ref34] Song Y. Sohl-Dickstein J. Kingma D. P. Kumar A. Ermon S. Poole B. (2021) Score-based generative modeling through stochastic differential equations. Available online at: https://arxiv.org/abs/2011.13456 (Accessed July 01, 2025).

[ref35] Too T. K. Li M. Y. (2016). A guideline of selecting and reporting intraclass correlation coefficients for reliability research. J. Chiropr. Med. 15, 155–163. doi: 10.1016/j.jcm.2016.02.012, 27330520 PMC4913118

[ref36] Xiang L. Qiao Y. Nie D. An L. Lin W. Wang Q. . (2017). Deep auto-context convolutional neural networks for standard-dose PET image estimation from low-dose PET/MRI. Neurocomputing 267, 406–416. doi: 10.1016/j.neucom.2017.06.048, 29217875 PMC5714510

[ref37] Yaakub S. N. McGinnity C. J. Clough J. R. Kerfoot E. Girard N. Guedj E. . (2019). “Pseudo-normal PET synthesis with generative adversarial networks for Localising Hypometabolism in epilepsies,” in Simulation and Synthesis in Medical Imaging, (Switzerland: Springer International Publishing), 42–51.

[ref38] Yan S. Wang C. Chen W. Lyu J. (2022). Swin transformer-based GAN for multi-modal medical image translation. Front. Oncol. 12:942511. doi: 10.3389/fonc.2022.942511, 36003791 PMC9395186

[ref39] Zhang Q. Zhou C. Zhang X. Fan W. Zheng H. Liang D. . (2024). Realization of high-end PET devices that assist conventional PET devices in improving image quality via diffusion modeling. EJNMMI Phys. 11:103. doi: 10.1186/s40658-024-00706-3, 39692956 PMC11656007

