# Supplementary material for: Score-based generative diffusion models to synthesize full-dose FDG brain PET from MRI in epilepsy patients
Source: Front Artif Intell. 2026 Jun 16;9:1841677. doi: 10.3389/frai.2026.1841677 (PMC13316877; doi:10.3389/frai.2026.1841677)
Supplement: Supplementary file 1 [file Supplementary_file_1.docx]

**Supplementary Materials**

**Training Hyperparameters**

**TransUNet.** The model was trained for 50 epochs using the Adam optimizer with a learning rate of 2 × 10⁻⁴ and weight decay of 1 × 10⁻⁴. A polynomial learning rate decay (PolyLR) scheduler was applied.

**SGM-KD.** The model consists of 3 blocks with depth 2. The noise scale parameters were set to σ_data = 0.5, σ_min = 0.01, and σ_max = 10, with a cosine-interpolated sigma sampling strategy. Training was performed for 100 epochs using the AdamW optimizer with a learning rate of 5 × 10⁻⁴ and weight decay of 1 × 10⁻³.

**SGM-VP.** The variance-preserving (VP) diffusion model was trained with β_min = 0.1 and β_max = 5 for 100 epochs. The learning rate was set to 1 × 10⁻⁴.

**Supplementary Table 1: ROI - Destrieux regions Mapping**

| ROI Name | Lateralized | Labels included |
| --- | --- | --- |
| Frontal cortex | Yes | G_and_S_frontomargin  G_and_S_transv_frontopol  G_front_inf-Opercular  G_front_inf-Triangul  G_front_middle  G_front_sup  S_front_inf  S_front_sup |
| Temporal cortex | Yes | G_oc-temp_lat-fusifor  G_oc-temp_med-Lingual  G_oc-temp_med-Lingual  G_temp_sup-G_T_transv  G_temp_sup-Lateral  G_temp_sup-Plan_polar  G_temp_sup-Plan_tempo  G_temporal_inf  G_temporal_middle  Pole_temporal  S_oc-temp_lat  S_oc-temp_med_and_Lingual  S_temporal_inf  S_temporal_sup  S_temporal_transverse |
| Parietal cortex | Yes | G_pariet_inf-Angular  G_pariet_inf-Supramar  G_parietal_sup  S_intrapariet_and_P_trans  S_subparietal |
| Occipital cortex | Yes | G_and_S_occipital_inf  G_occipital_middle  G_occipital_sup  Pole_occipital  S_occipital_ant |
| Insular cortex | Yes | G_Ins_lg_and_S_cent_ins  G_insular_short  S_circular_insula_ant  S_circular_insula_inf  S_circular_insula_sup |
| Cerebral white matter | Yes | Cerebral-White-Matter |
| Deep gray matter | Yes | Thalamus-Proper  Caudate  Putamen  Pallidum |
| Hippocampus + amygdala | Yes | Hippocampus  Amygdala |
| Brain stem | No | Brain-Stem |
| CSF | No | CSF |
| Corpus callosum | No | CC_Posterior  CC_Mid_Posterior  CC_Central  CC_Mid_Anterior  CC_Anterior |
| Cerebellum | Yes | Cerebellum-White-Matter  Cerebellum-Cortex |

**Supplementary Table 2: Subject Clinical Reports (Summarized)**

| Subject | Normal/Abnormal | Report |
| --- | --- | --- |
| **1** | **Abnormal** | Asymmetrically diminished FDG in left mesial temporal to left hippocampus |
| 2 | Normal | No definite area of abnormal hypo/hyper metabolism |
| **3** | **Abnormal** | Hypometabolism along right, mid lateral of temporal lobe along polymicrogyria |
| **4** | **Abnormal** | Hypometabolism in left mesial temporal lobe |
| 5 | Normal | Subtly diffusely decreased in uptake in left temporal |
| **6** | **Abnormal** | Focal hypometabolism seen in the anterior left temporal lobe |
| **7** | **Abnormal** | Diffuse asymmetric uptake in left temporal lobe when compared to the right |
| 8 | Normal | No focal hypometabolism to suggest a site of seizure focus |
| 9 | Normal | No evidence of seizure focus |
| 10 | Normal | No indication of abnormalities or normality in the report |

**Supplementary Table 3: Fourier Transform Table**

| **Model Type** | **Inputs** | **Mean difference for log-scale high frequency power (>0.2 cycles/pixel)** |
| --- | --- | --- |
| SGM-VP | T1w | -0.813 |
| SGM-VP | T1w, 1% | -0.690 |
| SGM-VP | T1w, T2F | -0.797 |
| SGM-VP | T1w, T2F, 1% | -0.639 |
| SGM-KD | T1w | -0.365 |
| SGM-KD | T1w, 1% | -0.212 |
| SGM-KD | T1w, T2F | -0.348 |
| SGM-KD | T1w, T2F, 1% | -0.297 |
| TransUnet | T1w | -0.066 |
| TransUnet | T1w, 1% | 0.086 |
| TransUnet | T1w, T2F | -0.164 |
| TransUnet | T1w, T2F, 1% | 0.122 |

Note: Negative differences indicate more high frequency power and more interslice inconsistencies in the slice direction.

**Supplementary Figures**

**Normal vs. Abnormal CI/CMAE/SUVR**

**
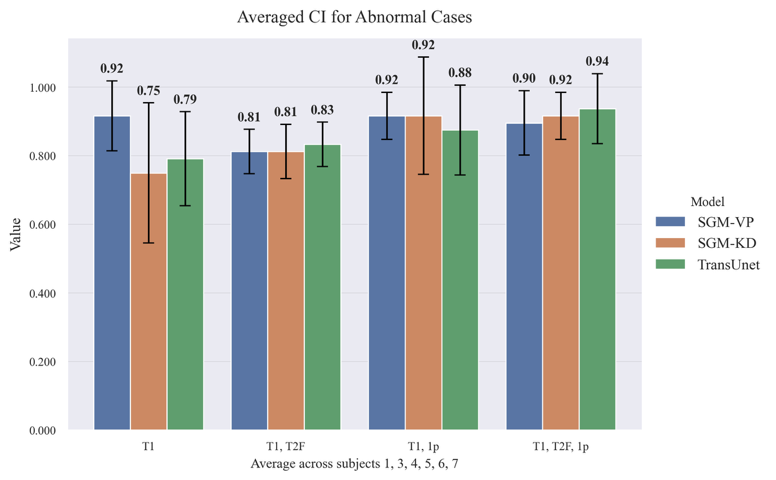

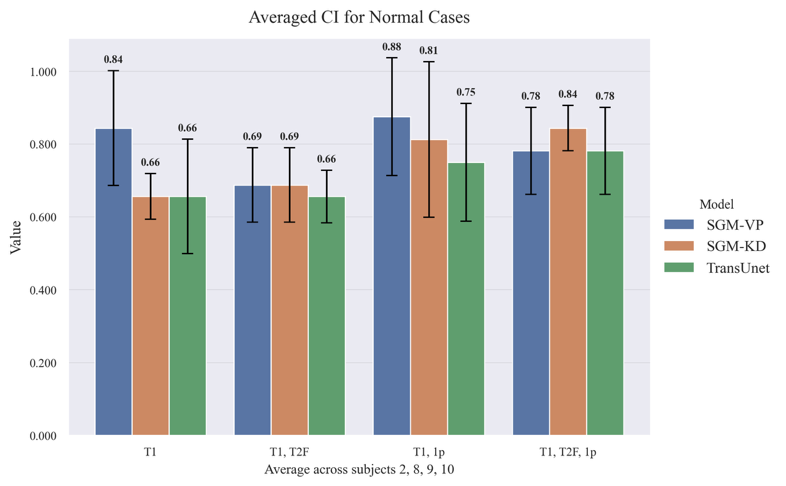
**

**Supplementary Figure 1:** CI in abnormal and normal groups


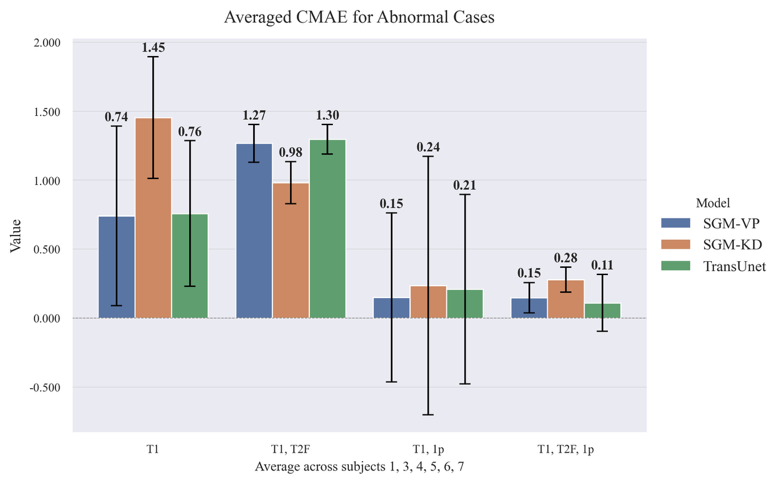
**
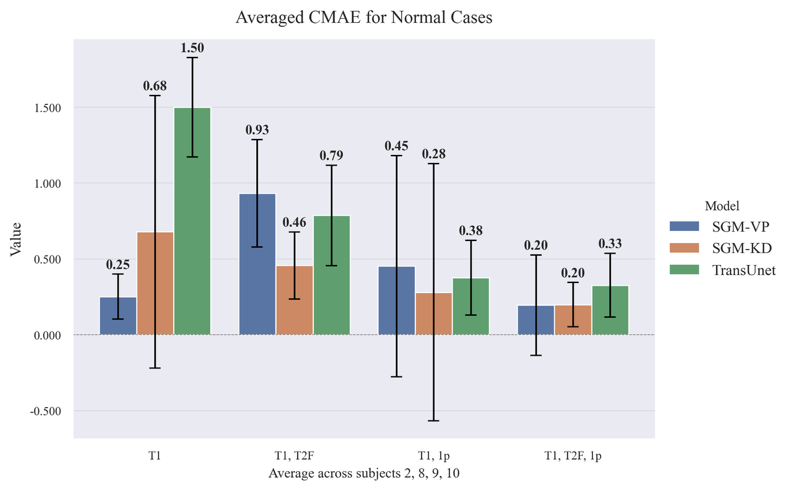
**

**Supplementary Figure 2:** CMAE in abnormal and normal groups (scaled by 1000).

**
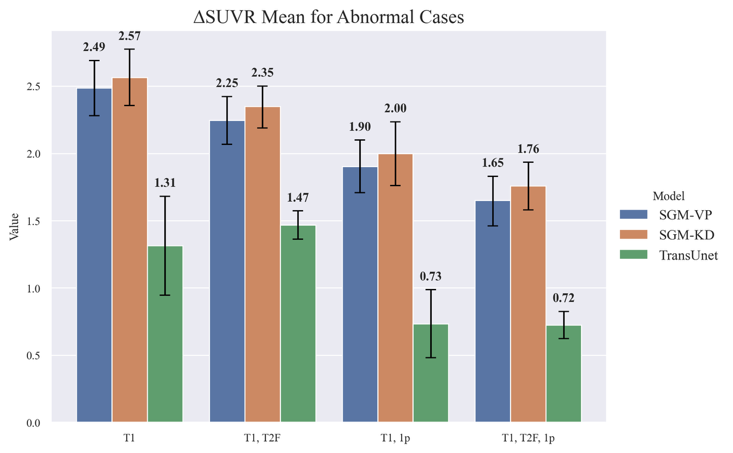

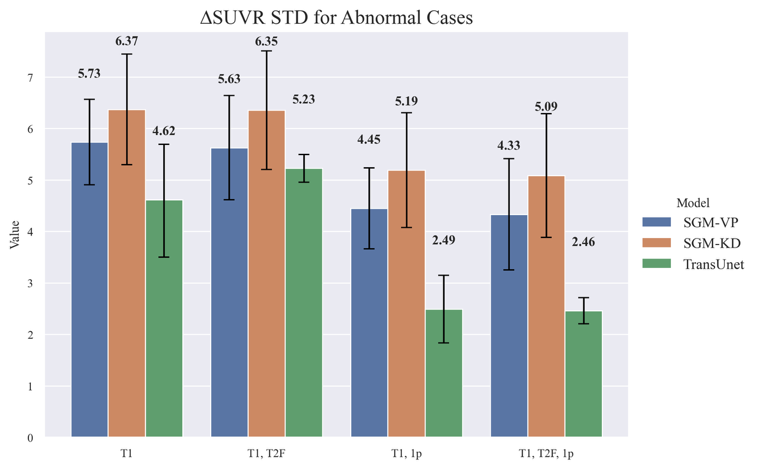
**

**
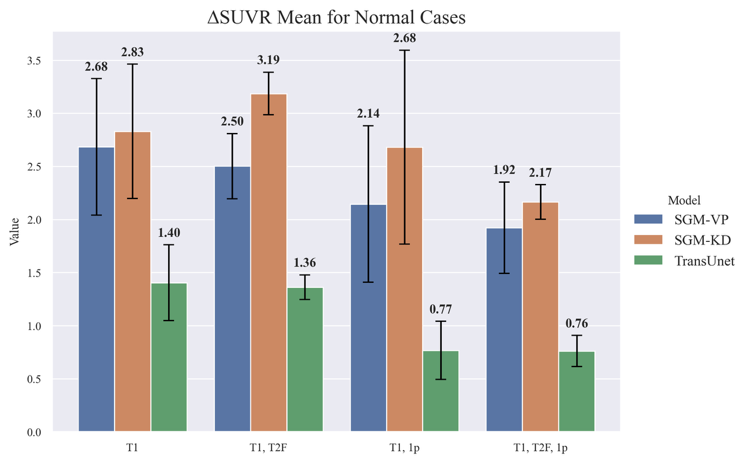

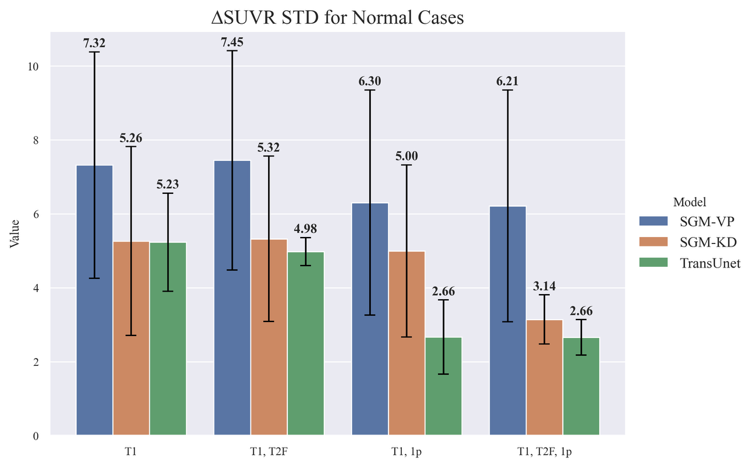
**

**Supplementary Figure 3:** $\Delta$SUVR Mean & STD in normal and abnormal groups
